# Laparoscopic Cortical Sparing Adrenalectomy for Pediatric Bilateral Pheochromocytoma: Anesthetic Management

**DOI:** 10.5812/aapm.15460

**Published:** 2014-04-07

**Authors:** Geetha Chamanhalli Rajappa, Tejesh Channasandra Anandaswamy

**Affiliations:** 1 Department of Anesthesiology, Rajiv Gandhi University of Health Sciences, Bangalore, India

**Keywords:** Pediatrics, Pheochromocytoma, Laparoscopy, Adrenalectomy, Anesthetics

## Abstract

**Introduction:**

Pheochromocytoma is a catecholamine-secreting tumor, which is seen rarely in children. These tumors predominantly secrete norepinephrine and epinephrine. They might be familial and associated with hereditary tumors such as Von Hippel-Lindau syndrome and multiple endocrine neoplasia type II.

**Case Presentation:**

The child might present with a spectrum of clinical manifestation including hypertension, headache, visual disturbances, and behavioral problems. A meticulous preoperative preparation is essential for a stable intraoperative and postoperative outcome

**Conclusions:**

We described successful perioperative management of a child who underwent bilateral laparoscopic cortical sparing adrenalectomy and a repeated surgery for the residual tumor removal.

## 1. Introduction

Pheochromocytoma and paraganglionoma are neuroendocrine tumors arising from chromaffin cells. Pediatric pheochromocytomas, although rare, have an increased incidence of bilateral, multifocal, and familial preponderance when compared to adults (1). It occurs in less than 2% of pediatric patients with hypertension and is a diagnosis of exclusion. Perioperative management of pheochromocytoma is challenging and requires multidisciplinary approach for optimal care and successful outcome.

## 2. Case Presentation

A 12-year-old 25 Kg male child referred to us for bilateral cortical sparing laparoscopic adrenalectomy. He complained of pain abdomen for six months, reduced appetite, and easy fatigability. General physical examination and systemic examination findings were unremarkable except for a high blood pressure of more than 160/100 mmHg for which oral nifedipine 5 mg (Depin, Zydus Cadila) twice daily was started and further evaluation was pending.

**Figure 1.**
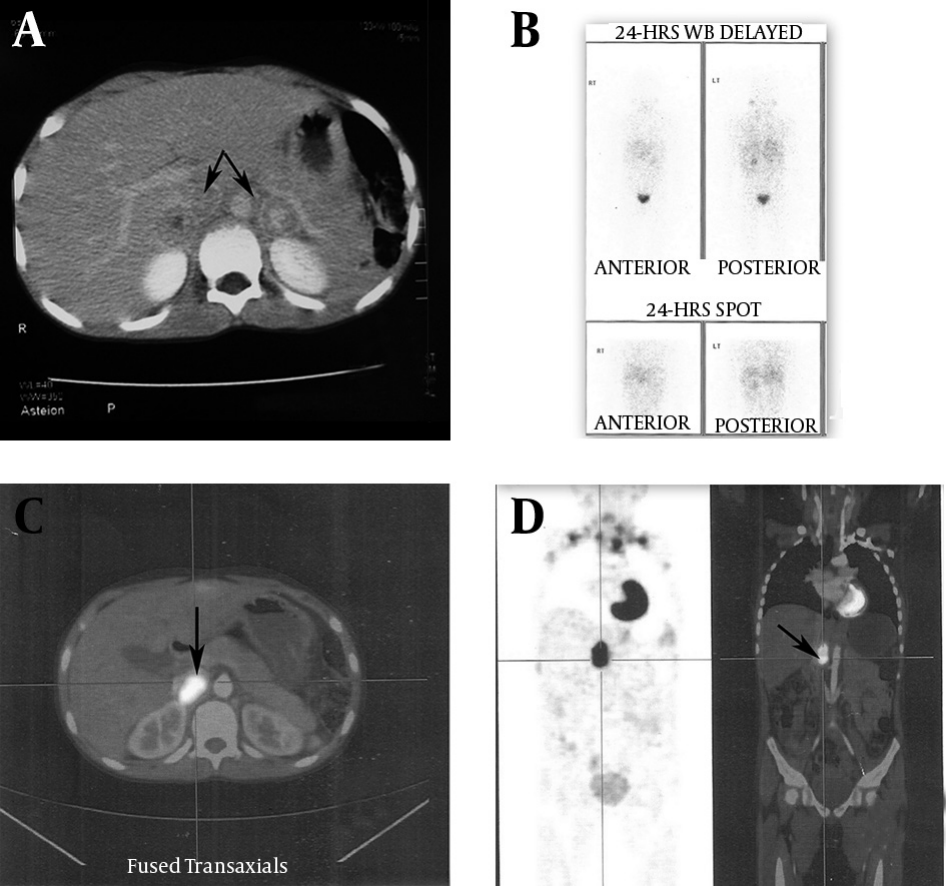
A: CT Scan of Abdomen Revealed Bilateral Pheochromocytoma, B: [^123^] I-Labelled MIBG Scan Showing Inconclusive Findings, C and D: PET Scan Revealed Metabolically Active Enhancing Nodule in the Right Suprarenal Region

On further investigations including complete blood counts, serum calcium, electrolytes, urea, and echocardiography (ECHO) results were normal. Ultrasound examination (USG) of the abdomen showed multiple heterogeneous bilateral adrenal lesions that were confirmed on computed tomography (CT) scan (Figure 1). Suspicion of bilateral pheochromocytoma was confirmed when 24 hr urinary normetanephrine was markedly elevated (4.424 mcg/day; Normal 0-600 mcg/day). Blood pressure prior to planned surgery was stabilized with prazosin (Prazopress, Sun phrama) 10 mg/day (2 mg-3 mg-2 mg-3 mg), nifedipine 5 mg Bid, and atenolol (Adbeta, Zuventus) 12.5 mg Bid. Volume expansion was achieved by increased oral salt intake and intravenous fluids during three days prior to surgery.

The child was premedicated with midazolam 1 mg (Midosed, Sun Pharma) intravenously and was transferred to the operating room on an infusion of normal saline 60 mL/hr and hydrocortisone 10 mg/hr (Cort-S, Neon Laboratories Ltd) according to the endocrinologist’s advice. After instituting electrocardiogram (ECG), noninvasive blood pressure (NIBP), and pulse oximeter (SpO_2_) monitors, the child was pre-oxygenated and anesthesia was induced with propofol 2 mg/Kg (Diprivan, AstraZeneca) and fentanyl 2 mcg/Kg (Verfen, Verve Health Care Ltd). Endotracheal intubation was facilitated with vecuronium 0.1 mg/Kg (Norcuron, Organon). Left subclavian vein and left radial artery were cannulated for continuous invasive pressure monitoring. An 18G epidural catheter was inserted in T8-T9 interspace for analgesia. Anesthesia was maintained with isoflurane 0.5-1.5% in air-oxygen mixture. Intraoperative blood pressure surge during the tumor dissection was controlled with titrated infusion of esmolol (Esocard, Samarth Life Sciences Ltd) and sodium nitroprusside (SNP) (Pruside, Troikaa). Estimated blood loss was about 300 mL, which was replaced by crystalloids to maintain adequate urine output throughout the procedure.

Paradoxically, the child still required SNP infusion for control of hypertension post bilateral adrenalectomy. Postoperatively, the child was ventilated and transferred to PICU with SNP infusion. Analgesia was managed with epidural infusion of 0.0625% bupivacaine (Anawin, Neon Laboratories Ltd) with 2 mcg/mL of fentanyl at 5 mL/hr. The child also received supplemental doses of intravenous morphine. Persistent hypertension postoperatively required SNP infusion initially and subsequently. The child received prazosin 1 mg sixth hourly, atenolol 12.5 mg OD, and nifedipine 5 mg Bid to control hypertension. Residual or extra-adrenal tumor was suspected in view of persistent hypertension and repeated 24 hr urine normetanephrines were 3480 mcg/day. As the metaiodobenzyl guanidine (MIBG) scan was inconclusive (Figure 1), a whole body positron emission tomography (PET) scan was ordered. PET scan revealed metabolically active enhancing nodule in the right suprarenal region that confirmed the suspicion of residual tumor (Figure 1). Hence, a repeat surgery via laparoscopic approach for removal of residual tumor was planned three weeks later.

During the second surgery the child was induced with thiopentone (Thiosol, Neon Laboratories Ltd) and endotracheal intubation was facilitated with atracurium. Right subclavian vein and left ulnar arteries were cannulated for invasive pressure monitoring. Epidural catheter was not employed since the child had refused placement. Anesthesia was maintained with isoflurane 0.5-1.5% in air-oxygen mixture. Blood pressure changes during tumor handling were controlled with titrated infusion of nitroglycerine (NTG) (Nitrovir, Chandra BhagatPharma). Following removal of residual tumor, NTG was rapidly tapered. The child was extubated and transferred to PICU. The child remained hemodynamically stable postoperatively and analgesia was achieved with intravenous paracetomol and morphine. Post-operative blood glucose level remained normal. Serum cortisol level examined two weeks later and the result was normal (13 mcg/dL).The child had an uneventful postoperative course and was subsequently discharged. In the follow up after three months, the child had normal blood pressure and serum cortisol level.

## 3. Discussion

Pheochromocytoma in children accounts for 10-20% of the diagnosed cases, presenting at ten to 12 years of age with a male preponderance. They may be familiar and there is an association with hereditary tumor syndrome like Von Hippel-Lindau syndrome and Multiple Endocrine Neoplasia Type 2 (MEN 2). Clinical presentation range from being asymptomatic to symptoms of sustained hypertension like headache, sweating, visual problems, nausea, and vomiting. It may also present with decreased school performance or behavioral problems (2). Measurements of plasma and/or urinary metanephrines are the most reliable biochemical tests available, with almost 100% sensitivity (3). Measurement of homovanillic acid (HVA) and vanillylmandelic acid (VMA) might give false positive results. Imaging studies like CT scan and magnetic resonance imaging (MRI) provide precise tumor localization. However, paraganglionoma and recurrent tumors are better confirmed by functional imaging by [^123^] I-labelled MIBG scintigraphy. Presently, functional imaging study by [^18^F] fluorodopamine (FDA) PET is superior to MIBG in evaluation of malignant chromaffin cell tumors (4).

Surgical resection is the treatment of choice for bilateral pheochromocytoma. Both open and laparoscopic approaches have been successfully employed depending on the surgeon’s choice and skill. In case of bilateral adrenalectomy, post-operative glucocorticoid and mineralocorticoid replacement is necessary. A Cortical sparing adrenalectomy is advantageous in patients with bilateral disease to avoid long-term glucocorticoid deficiency, especially in children (2). Postoperative cortisol levels have to be monitored periodically as we performed in our patient. The fundamental goals of preoperative medical therapy are optimal control of blood pressure, heart rate, and function of other organs. In addition, it is necessary to restore blood volume and to impede intraoperative catecholamine surges with its consequent cardiovascular effects. Wide variety of protocols and regimens for preoperative optimization exist, but there is lack of evidence-based studies to compare them (5).

Conventionally, the patients are started on alpha-adrenergic blockers for ten to 14 days prior to surgery. Alpha blockade is usually achieved with noncompetitive blocker phenoxybenzamine 0.2-1 mg/Kg/day in divided doses (2), which might offer the theoretical advantage over competitive blocker in case of catecholamine surge. Alternatively, selective alpha blockers like prazosin or doxazocin are titrated to achieve rapid effect and their shorter duration might prevent prolonged hypotension post-operatively (5). Calcium channel blockers might be used if alpha blockers are ineffective or in case of intolerable side effects (1). Metyrosine, atyrosine hydroxylase inhibitor that blocks catecholamine synthesis, has also been used (1, 6). Subsequently, only after achieving adequate alpha blockade the beta blockade therapy has to be instituted to counteract any secondary tachycardia and tachyarrhythmia (5, 7). Non-selective beta-blockers like propranolol and selective beta-1 blockers like atenolol, metoprolol, or biosprolol can be used. Blood volume expansion with oral salt replacement (2) and/ or intravenous saline infusion is recommended to reduce postoperative hypotension.

Preoperative benzodiazepine and reassurance reduces anxiety and pressure fluctuations. Various anesthetic techniques have been employed successfully. Combined epidural and general anesthesia is the most preferred anesthesia technique (8). Standard monitors like ECG, NIBP, and SPO_2_ are mandatory before induction of anesthesia. Invasive arterial pressure and central venous pressure monitoring are essential and are ideally employed after anesthesia induction in a pediatric patient. Temperature and urine output should also be monitored. Transesophageal echocardiography is recommended in cases of catecholamine induced cardiomyopathy or intracardiac pheochromocytoma (7). Thiopentone or propofol (8) can be used for induction of anesthesia along with fentanyl and both have a good hemodynamic profile. Vecuronium is the preferred muscle relaxant for cardiovascular stability but atracurium and rocuronium have been used without any untoward effect. Epidural infusion of bupivacaine 0.1-0.125% with fentanyl 2 mcg/mL at the rate of 5-10 mL/hr can be used for post-operative analgesia (8).

Intraoperative hypertensive spikes during induction and tumor manipulation can be impeded by deepening anesthesia and infusions of SNP, NTG, phentolamine, esmolol, nicardipine, dexmedetomidine, or magnesium sulphate. A single drug or combination regimens might be employed depending on severity, familiarity, and availability (7-9). We used different induction agents, muscle relaxants, and drugs to counter intraoperative pressure rises, as the anesthetic teams were different for the two procedures. Post resection, patients should be monitored for complications of hypotension, hypoglycemia, and persistent hypertension. Hypotension can be severe, and might need phenylephrine, adrenaline or noradrenaline (7), or vasopressin infusions (10), especially in the patients receiving phenoxybenzamine. Hypertension might persist for few days due to elevated circulating catecholamines. Hypoglycemia might ensue after removal of tumor, secondary to increase in insulin levels due cessation of pancreatic beta cell suppression (11). Decision for post-operative extubation or elective ventilation depends on hemodynamics stability and other vital parameters. Post-operative ICU care is necessary for close monitoring of the complications. In the event of persistent hypertension beyond 7-10 days, presence of residual tumor (12) or extra adrenal tumor should be considered. Biochemical assay and imaging studies are repeated for confirmation and further management. Malignant tumors and metastatic disease which are not amenable for surgery can be treated with palliative modalities like [^131^I] MIBG, somatostatin analogs, and chemotherapy for symptomatic relief and tumor regression (2). Pediatric pheochromocytomas are rare, can be multifocal, and might have genetic predisposition. Newer biochemical assays and imaging studies aid in accurate diagnosis and localization of the tumor. Laparoscopic cortical sparing adrenalectomy is the preferred treatment in children with bilateral tumors. A multidisciplinary approach team comprising of anesthesiologists, surgeon, endocrinologists, and cardiologists is vital for successful outcome.
